# Design of a Computer Model for the Identification of Adolescent Swimmers at Risk of Low BMD

**DOI:** 10.3390/ijerph20043454

**Published:** 2023-02-16

**Authors:** Jorge Marin-Puyalto, Alba Gomez-Cabello, Alejandro Gomez-Bruton, Angel Matute-Llorente, Sergio Castillo-Bernad, Gabriel Lozano-Berges, Alejandro Gonzalez-Agüero, Jose A. Casajus, German Vicente-Rodriguez

**Affiliations:** 1 GENUD “Growth, Exercise, Nutrition and Development” Research Group, Universidad de Zaragoza, 50009 Zaragoza, Spain; 2 Department of Physiatry and Nursing, Faculty of Health Sciences (FCS), Universidad de Zaragoza, 50009 Zaragoza, Spain; 3 Centro Universitario de la Defensa, 50090 Zaragoza, Spain; 4 Instituto Agroalimentario de Aragón-IA2, Universidad de Zaragoza-CITA, 50009 Zaragoza, Spain; 5 Centro de Investigación Biomédica en Red de Fisiopatología de la Obesidad y Nutrición (CIBERObn), 28029 Madrid, Spain; 6 Department of Physiatry and Nursing, Faculty of Health and Sport Sciences (FCSD), Universidad de Zaragoza, 22001 Huesca, Spain

**Keywords:** osteoporosis prevention, decision tree, physical fitness, screening

## Abstract

This paper aims to elaborate a decision tree for the early detection of adolescent swimmers at risk of presenting low bone mineral density (BMD), based on easily measurable fitness and performance variables. The BMD of 78 adolescent swimmers was determined using dual-energy X-ray absorptiometry (DXA) scans at the hip and subtotal body. The participants also underwent physical fitness (muscular strength, speed, and cardiovascular endurance) and swimming performance assessments. A gradient-boosting machine regression tree was built to predict the BMD of the swimmers and to further develop a simpler individual decision tree. The predicted BMD was strongly correlated with the actual BMD values obtained from the DXA (r = 0.960, *p* < 0.001; root mean squared error = 0.034 g/cm^2^). According to a simple decision tree (74% classification accuracy), swimmers with a body mass index (BMI) lower than 17 kg/m^2^ or a handgrip strength inferior to 43 kg with the sum of both arms could be at a higher risk of having a low BMD. Easily measurable fitness variables (BMI and handgrip strength) could be used for the early detection of adolescent swimmers who are at risk of suffering from low BMD.

## 1. Introduction

Osteoporosis is a metabolic disease that is characterized by a deterioration in skeletal tissue, including a clinically low bone mineral density (BMD) and a compromised microarchitecture of the bone [1]. Osteoporosis affects 22.1% of women and 6.6% of men over 50 years of age in the European Union, and the total number of patients with this condition is expected to increase in the following years due to demographic variations [2]. This structural fragility entails a lower tolerance to stress, which may play a role in up to 90% of bone fractures [3]. These osteoporotic fractures have been linked to a decrease in the quality of life, the apparition of disabilities, and even mortality [4]. Nowadays, osteoporosis treatment and prevention have become one of the primary concerns for healthcare systems in developed countries [5]. In fact, a total of 33 different clinical practice guides on osteoporosis screening and management issued by institutions all around the globe have been identified and evaluated in a recent systematic review [6], concluding that collaboration and consensus are needed in the elaboration of these guidelines.

Adolescence stands as a decisive period for osteoporosis prevention since around 40% of adult bone mass is created during this stage [7] and will influence the peak BMD that is reached in early adulthood. Achieving the highest peak BMD possible is key for the prevention of osteoporosis later in life [7], given that fracture risk is expected to be halved with an increase of one standard deviation in the peak bone mass [8].

Physical activity participation and calcium intake are among the controllable factors that are known to affect bone health during childhood and adolescence [9]. Different reviews focused on sports practice [10] and exercise interventions [11] have found consistent and positive effects of these on bone development. Additionally, the benefits of physical activity on bone status have been shown to persist in later stages of life [12]. However, not all sport modalities have proven to be beneficial to bones, especially non-weight-bearing activities, such as swimming, which may have a neutral [13] or even negative [14] effect on bone health despite their positive effect on muscle mass.

Osteoporosis has been defined as a “silent” disease, given that no pain or other symptoms are perceived by the subject who is affected by this condition [3]. For this reason, osteoporosis and low BMD can remain undetected for years, and, sometimes, they are only discovered once an osteoporotic fracture has occurred. Thus, there are subjects who are unknowingly affected by this condition, unaware of their higher risk of fractures, and, while this condition remains unnoticed, no preventive measures could be taken.

The early detection of subjects affected by a low BMD is, therefore, of paramount importance. A low birth weight has been proposed as a risk factor for low BMD during adulthood [15], but after studying monozygotic twins with different birth weights, Frost et al. [16] concluded that the differences in adult BMD are more likely due to the differences in body size rather than in bone metabolism. Public health recommendations include an osteoporosis screening for the elderly [17] using the current gold standard method for BMD evaluation, which is dual-energy X-ray absorptiometry (DXA). However, this clinical evaluation is rarely performed in other population segments that might also be susceptible to suffering from decreased BMD, such as swimmers.

Correctly identifying a target group for BMD assessments is important regarding the cost-effectiveness of osteoporosis management [18]. However, performing DXA scans on all adolescent swimmers would not be cost-effective, especially taking into account that the younger the target group, the higher the number of scans needed to prevent one fracture [19]. For this reason, making the assessment of different variables related to BMD more accessible to researchers or healthcare workers may provide a tool for determining which subjects might need a deeper evaluation of their bone mineral status.

Therefore, the main goal of the present study is to elaborate a screening method for detecting potential risks of low BMD in adolescent swimmers based on easily measurable variables. The authors hypothesized that low physical fitness values, mainly muscular strength and body composition, would increase the risk of suffering from low BMD in adolescent swimmers due to the fact that poorer values in these parameters could negatively affect bone development.

## 2. Materials and Methods

### 2.1. Participants and Study Design

A total of 86 adolescent swimmers (41 females, all Caucasian, and aged 10–18 years) participated in the present cross-sectional study, which is part of the broader RENACIMIENTO project [20]. The participants had to have a minimum of 3 years of regional swimming competition experience and train at least 6 h per week in order to be part of the study. The exclusion criteria included the following: smoking, taking medication that is known to alter bones, and suffering from chronic diseases or musculoskeletal disorders. The participants, researchers, and statisticians were not blinded.

Written informed consent was obtained from parents, and all of the participants expressed their agreement. The protocol study was approved by the Ethics Committee of Clinical Research from the Government of Aragón (ref. CP08/2012; CEICA, Zaragoza, Spain), and the ethical guidelines for human research outlined in the Declaration of Helsinki (revision of Seoul 2008) were followed.

### 2.2. Anthropometric and Bone Measurements

The participants underwent an anthropometric examination while wearing no shoes and minimal clothing. Their height was measured with a stadiometer to the nearest 0.1 cm (SECA 225, SECA, Hamburg, Germany), and their weight was measured to the nearest 0.1 kg with an electronic scale (SECA 861, SECA, Hamburg, Germany). Their body mass indices (BMIs) were calculated as their weight (kg) divided by their squared height (m^2^).

The bone mineral content (g) and areal BMD (g/cm^2^) were determined by means of a DXA scan of the whole body and the hip, evaluated with the pediatric version of the QDR-Explorer software, version 12.4 (Hologic Corp., Bedford, MA, USA). All scans were performed by the same qualified operator, who had been trained in the operation of the scanner, the positioning of the subjects, and the analysis of the scans according to the manufacturer’s guidelines. The coefficients of variation for the DXA measurements in our laboratory have already been published [21] and were 2.3% for BMC and 1.3% for BMD.

The subtotal (whole body less head) BMD height-adjusted Z-scores were calculated according to the reference values provided by Zemel et al. [22]. A Z-score of −1 was used as the threshold value for the purpose of categorizing subjects at risk of suffering from low BMD in the future. It should be highlighted that Crabtree et al. defined low bone mass in the pediatric population as a BMD Z-score equal to or lower than −2. The subtotal whole body was used as it is one of the regions recommended by the International Society for Clinical Densitometry in pediatric populations [23]. A height adjustment is also advised in this official statement [23].

### 2.3. Evaluation of Pubertal Stage

Pubertal maturation was determined by self-assessment of secondary sexual characteristics with the assistance of a graphical scale, following the method established by Tanner [24], which has been demonstrated as a valid and reliable method to assess sexual maturity among adolescent athletes [25].

### 2.4. Fitness Assessment

Four different components of physical fitness were assessed using field tests. The strength of the upper limbs was determined by the sum of both arms in a maximum isometric handgrip strength test with a dynamometer (TKK 5101, Takei Corp., Tokyo, Japan), while the strength of the lower limbs was assessed by the standing long jump test. The running speed was calculated from the time to complete a 30 m sprint, and the aerobic endurance was assessed by means of a 20 m shuttle run test [26]. All tests were performed twice, and the best result from the attempts was recorded with the exception of the aerobic endurance test, which was performed once.

### 2.5. Performance and Questionnaires

The swimming history was acquired from a self-reported questionnaire in which the participants stated their weekly hours of training and their swimming competition experience (in years). The structured questionnaire also included information on current and past participation in other sports. Additionally, their swimming performance was obtained by obtaining official timings from swimming competitions and recording the participants’ times in 50 m freestyle and their International Swimming Federation (FINA) ranking points, an official metric used by the FINA to track swimmer performance. The daily calcium intake (in milligrams) was calculated from a food frequency questionnaire that included the daily, weekly, and monthly consumption frequency of various calcium-containing foods, such as cheese or bread [27], which has been validated for adolescent swimmers [28].

### 2.6. Statistical Analysis

The statistical analyses were performed using SPSS for Windows, version 22.0 (SPSS Inc., Chicago, IL, USA), with the significance level set at *p* < 0.05. Additionally, the construction of the decision tree was performed with the statistical programming language R (version 4.2.2) [29], including the packages *rpart* [30] and *gbm* [31].

Kolmogorov–Smirnov tests were used to confirm the normality assumption, and an outlier exploration was performed for all variables included in the study. *T*-tests for the independent samples were used to check the differences between the subjects with and without low BMD values on the fitness and performance variables.

#### Decision Tree Modeling

A total of 70% of the sample was randomly selected to build a decision tree in order to identify the fitness and performance variables that show better discrimination between the BMD groups, whereas the remaining 30% of the sample was used to test its classification accuracy. A cross-tabulation of sex and pubertal development status across the groups was performed, and chi-square statistics were used to determine the homogeneity of the group distribution of the categorical variables between the training and testing subsamples.

Two decision trees were constructed by following different approaches. The first one was a regression tree (treating the subtotal BMD as a continuous variable) fitting all measured variables. Gradient-boosting [32,33] was implemented to increase its precision. From this initial computer model, a second model was developed. This decision tree [34] included solely the nine variables that were significant in the previous model and considered only the following two possible outcomes: being above or below a Z-score of −1 for the subtotal BMD. The performance of the models was evaluated by calculating the Pearson’s correlation coefficient and the Lin’s concordance coefficient for the regression tree and the out-of-bag error for the decision tree.

A summary of the variables included in the decision tree modeling is provided in Table S1. The code used for the construction of both models can be found in Document S1.

## 3. Results

### 3.1. Participant Characteristics

After removing participants with incomplete or outlier data, 78 participants out of the total sample of 86 swimmers were analyzed. Table 1 presents their descriptive characteristics, stratified according to their random allocation to the training or testing subsample. No differences between the groups were found for any studied variable.

The results from the fitness and performance comparison between the participants above and below the threshold in the total subtotal BMD Z-score are shown in Table 2. Differences were found between the groups for handgrip strength, long jump, 50 m swim, and FINA points (all *p* < 0.05).

### 3.2. Gradient-Boosting Machine Regression Tree

**Figure 1:**
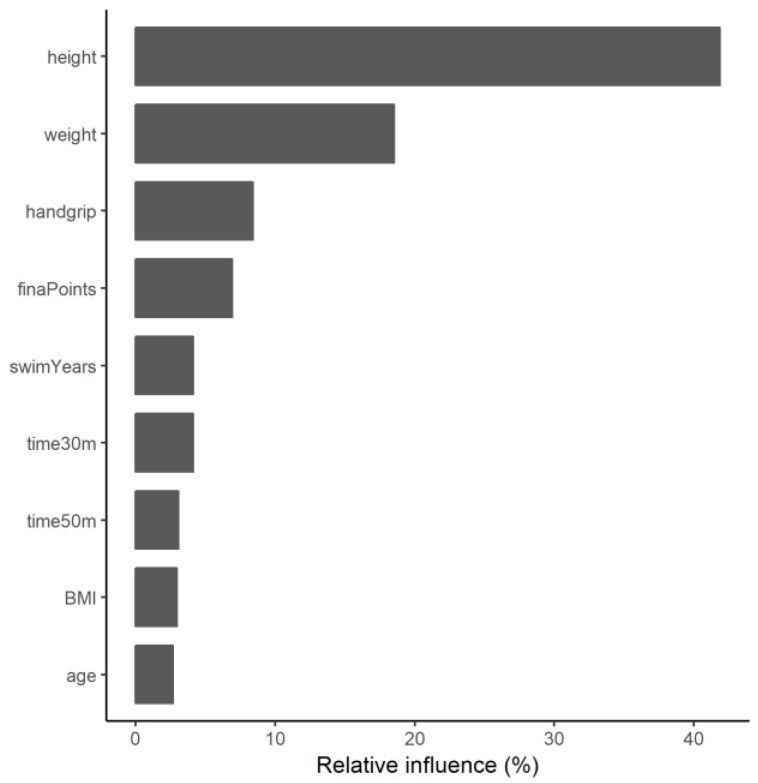
Relative contribution of the main variables of the ensemble model.

After scouting and tuning hyper-parameters for the gradient-boosting machine (Document S1), the optimal robustness was found using a total of five iterations of the gradient-boosting model. Nine of the variables included in the model had a significant influence. An overview of the significant variables is provided in Figure 1, where it can be observed that height and weight are the variables that contribute the most to the model’s prediction (41.9 and 18.5%, respectively), followed by handgrip strength (8.4%).

**Figure 2:**
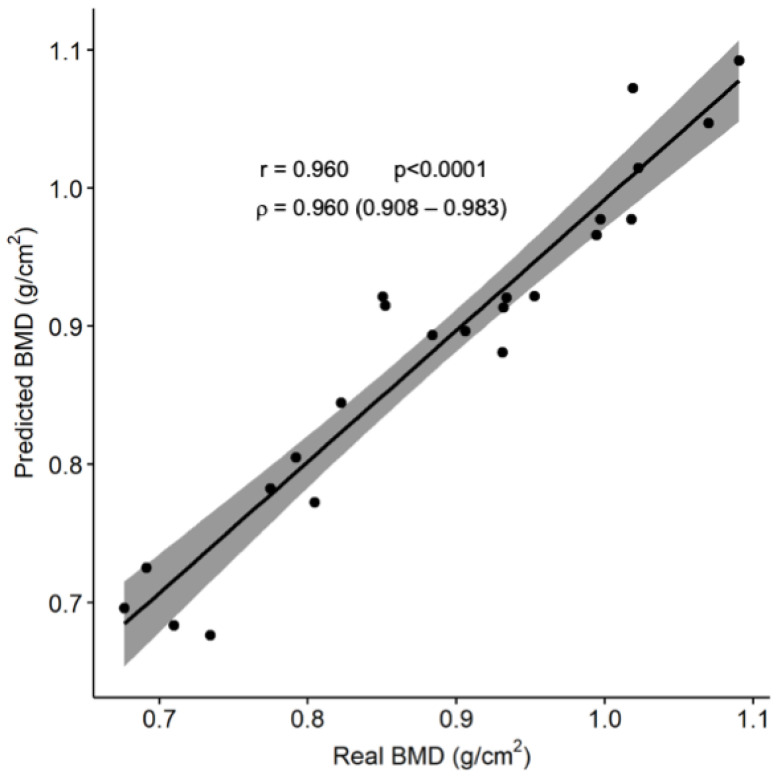
Comparison between the predicted and actual subtotal BMD.

When this computer regression model was applied to the 30% of the sample that was intended to test its prediction accuracy, a root mean squared error of 0.034 g/cm^2^ was obtained. The BMD values obtained from the model were significantly correlated with the actual BMD values as measured by the DXA (r = 0.960; ρ = 0.960; *p* < 0.001; Figure 2). When converted into the corresponding height-adjusted Z-scores, a 74% classification accuracy was reached when comparing the participants above and below the proposed threshold.

The solid line represents the regression function between the variables, while the gray area shows its 95% confidence interval. Abbreviation: BMD: bone mineral density.

### 3.3. Individual Decision Tree

**Figure 3:**
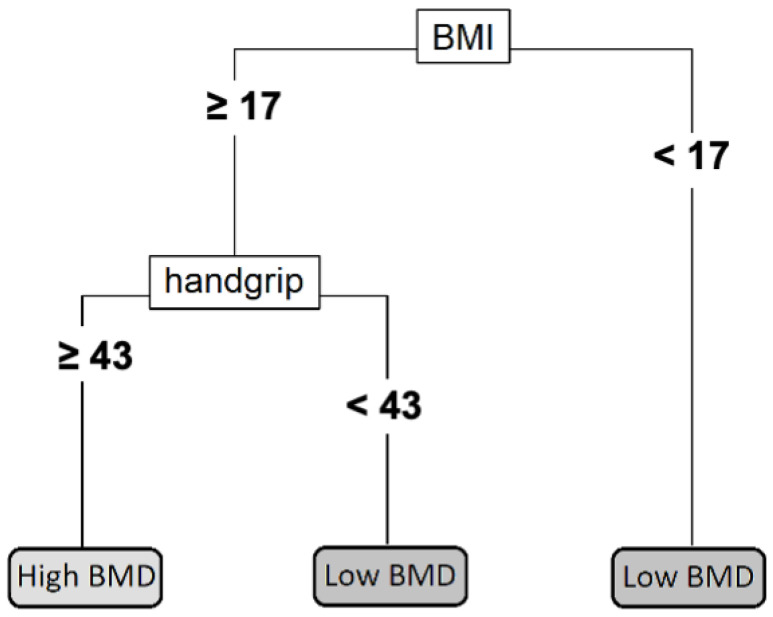
Individual decision tree. The handgrip strength is measured in kg by adding the results from both arms. The BMI is measured in kg/m^2^. Abbreviations: BMI: body mass index; BMD: bone mineral density.

Figure 3 shows the development of a single classification tree that splits the training sample into four terminal nodes. According to this model, subjects who have either a BMI under 17 kg/m^2^ or less than 43 kg of handgrip strength (summing both arms) present a higher risk of having low subtotal BMD for their age and height. This individual decision tree has an overall classification accuracy of 74%, a sensitivity of 50%, and a specificity of 82%.

## 4. Discussion

The main results from the present document are that physical fitness and performance variables can be used for the prediction of low BMD in adolescent swimmers and to establish appropriate further body composition examinations.

The relevance of the present study is the development of a practical tool for an initial screening of adolescent swimmers at risk of suffering from low BMD. Swimming trainers or healthcare workers can easily measure all the variables included in the model without demanding material or human resources. Most variables can be measured with the help of a questionnaire or a chronometer; the main exception is the handgrip test, which requires a dynamometer. However, the use of this test is justified for the following three reasons: it has been related to other health and performance parameters [35,36], it contributes significantly to both the regression and decision models, and it is a particularly quick, cheap, and simple test.

Regarding the specific results of the gradient-boosting machine model, the height and weight of the participants were the variables that affected their BMD the most. However, some physical fitness and performance variables accounted for some of the model’s prediction, especially handgrip strength. The mean squared error of the model did not result in a systematic or proportional bias in the prediction of the total subtotal BMD, which is further supported by the high concordance coefficient obtained. The classification accuracy of 0.739 is not perfect, but it is better than chance alone and similar to the areas under the curve (AUC) reported [37] for other screening strategies devised for postmenopausal women, such as the FRAX tool [38] (AUC: 0.60), the Simple Calculated Osteoporosis Risk Score [39] (AUC: 0.72), and the Osteoporosis Self-Assessment Tool [40] (AUC: 0.73).

These results are obtained from the model that includes all sixteen variables in a gradient-boosting machine and can provide interesting theoretical information. However, this model might not be easy to implement since it requires a high number of measurements and a computer evaluation of the model. In order to offer a useful tool for trainers that might not have the time and skills needed to perform the complete evaluation, an individual decision tree is provided that is based solely on the handgrip test and BMI measurement.

In the particular case of our testing sample, three out of the six participants that actually had a subtotal BMD Z-score below −1 would have been recommended to undergo a DXA scan based on the results from our model. Additionally, only two of the other seventeen participants in the subsample would have been erroneously advised to have their BMD checked.

The accuracy and validity of both the gradient-boosting model and the individual decision tree have been assessed from the perspective of trying to simultaneously optimize both the sensitivity and the specificity of the model. However, decision tree analyses allow for penalizing false positive and false negative cases differently by assigning their specific costs. This could be interesting because it can be argued that, in this case, it might be preferable to have a healthy subject scanned rather than failing to identify a participant at risk of having low BMD. A cost-effective analysis of the osteoporosis prevention system could reveal the actual cost of a false negative over a false positive, which, in turn, would provide the optimal balance between specificity and sensitivity that the model should seek. However, doing so would require a specific evaluation of the costs of healthcare interventions, which may vary between countries and, therefore, limit the geographical applicability of the model.

It is important to note that the purpose of the presented models is not to provide clinical diagnostics or to replace the need for DXA scans in any way, but rather to complement them, serving as an early screening filter for detecting those subjects who would benefit the most from a DXA assessment. The cost-effectiveness of the osteoporosis screening protocol for postmenopausal women has been confirmed [18]. However, the final implementation of these protocols is not always comprehensive, and sensitization strategies have been implemented [41,42]. A previous study that evaluated the effects of the implementation of a temporary clinical case-finding strategy for osteoporosis detection in postmenopausal women showed a great improvement in the screening policy using a simple index based solely on the age and weight of the subject [43]. Additionally, model-based tools have been successfully implemented to assess fracture risks in the elderly in the framework of clinical osteoporosis management [38]. However, to the best of our knowledge, no similar studies have been performed with adolescent swimmers. We consider that this is of primary importance, given the popularity of this sport nowadays among young populations, and it will permit the early identification of individuals with low BMD and allow us to start taking action early and act preventively in order to avoid future cases of osteoporosis.

Some limitations have to be acknowledged as well. The reduced size and wide age range of the test subsample might require confirmation of the validity and adjustment of the model in specific populations. However, it should be pointed out that no differences in age or sex distribution were found between the testing and training subgroups or among the low and normal BMD groups. Additionally, even though fitness and nutrition variables were considered, there might be other variables that affect BMD that should be included in the regression algorithm. Another limitation is that this theoretical model only included Caucasian adolescent swimmers; thus, future studies should include other populations, such as non-Caucasian adolescents or non-athletic swimmers.

## 5. Conclusions

In conclusion, this study presents a theoretical model and a practical tool for the early detection of adolescent swimmers at risk of low BMD, which can be quickly applied by swimming trainers or health professionals as a first step in seeking clinical advice for those subjects that were previously unaware of their condition. Nevertheless, future research should confirm the applicability of the model in other samples and investigate if the addition of other easily measurable variables could improve the prediction accuracy.

It should be highlighted that swimmers at risk of having low BMD could have bone health problems in the future. To counteract this problem, it would be recommended to combine swimming with other weight-bearing activities or sports, which could provoke an adequate stimulus to increase their bone parameters during growth.

## Figures and Tables

**Table 1:** Descriptive characteristics of the participants.

Variables	Overall (*n* = 78)	Training (*n* = 55)	Testing (*n* = 23)
General characteristics and anthropometric variables			
Sex (male/female)	40/38	26/29	14/9
Tanner stage (I/II/III/IV/V)	2/18/16/38/7	1/10/13/27/4	1/6/3/11/2
Age (years)	14.3 ± 1.9	14.3 ± 1.8	14.3 ± 2.2
Height (cm)	163.8 ± 12.0	163.1 ± 11.3	165.4 ± 13.5
Weight (kg)	54.3 ± 12.1	53.3 ± 11.2	56.7 ± 13.9
BMI (kg/m^2^)	20.0 ± 2.5	19.8 ± 2.3	20.4 ± 2.8
Bone mineral variables			
Subtotal BMC (g)	1388 ± 416	1350 ± 392	1481 ± 465
Subtotal BMD (g/cm^2^)	0.867 ± 0.113	0.858 ± 0.108	0.889 ± 0.123
Total hip BMC (g)	29.9 ± 9.1	28.9 ± 8.2	32.1 ± 10.6
Total hip BMD (g/cm^2^)	0.888 ± 0.133	0.879 ± 0.136	0.907 ± 0.126

The categorical variables are expressed as frequencies, and the continuous variables are expressed as means ± standard deviations. Abbreviations: BMI: body mass index; BMC: bone mineral content; BMD: bone mineral density. No significant differences were found between the groups.

**Table 2:** Fitness and performance comparison between the subjects with and without low BMD.

Variables	Overall (*n* = 78)	Low BMD (*n* = 24)	Not Low BMD (*n* = 54)
Fitness			
Handgrip strength (kg)	53.5 ± 17.3	46.3 ± 16.1	56.7 ± 17.0 *
Long jump (cm)	185.6 ± 30.4	174.3 ± 31.8	191.0 ± 28.5 *
30-m run (s)	5.21 ± 0.48	5.33 ± 0.42	5.15 ± 0.50
VO_2max_ (mL/kg·min)	50.4 ± 5.3	49.3 ± 5.0	51.0 ± 5.3
Training and performance			
Weekly training (h)	10.0 ± 2.1	9.8 ± 2.1	10.0 ± 2.1
Swimming history (years)	7.9 ± 2.9	7.6 ± 2.7	8.0 ± 3.0
50-m swim (s)	31.2 ± 3.5	32.8 ± 4.4	30.5 ± 2.8 *
FINA points	354 ± 84	317 ± 82	370 ± 80 *

Values expressed as means ± standard deviations. Abbreviations: BMD: bone mineral density; VO_2max_: maximal oxygen uptake; FINA: International Swimming Federation. * Significant differences compared to the low BMD group.

## Data Availability

Not applicable.

## Supplementary Materials

The following supporting information can be downloaded at: https://www.mdpi.com/article/10.3390/ijerph20043454/s1, Table S1: Summary of the variables included in the regression and decision trees; Document S1: The code used for the construction of both models.
