# Supplementary material for: Design of a Computer Model for the Identification of Adolescent Swimmers at Risk of Low BMD
Source: Int J Environ Res Public Health. 2023 Feb 16;20(4):3454. doi: 10.3390/ijerph20043454 (PMC9964481; doi:10.3390/ijerph20043454)
Supplement: Supplementary file 1 [file ijerph-20-03454-s001.zip › Supplementary Document S1.pdf]

Document S1: The code used for the construction of both models

```
#loading necessary packages
library(dplyr)
library(rpart)
library(rpart.plot)
library(gbm)
library(ggplot2)
library(vip)
library(pdp)
library(boot)
library(ggpubr)

#loading data and isolating the variables included in model construction
load("RENACIMIENTO.RData")
treeDB<-renacimiento[,c(12,2:9,30:37)]

#for reproducibility
set.seed(1)

#randomly selecting 70% of the sample
random_index<-sample(1:nrow(treeDB),nrow(treeDB))
treeDB<-treeDB[random_index,]
train<-sample(1:78,round(78*0.7,0))

#creating a matrix to test different hyper-parameter values
hyper_grid <- expand.grid(
  shrinkage = c(.01, .1, .3),
  interaction.depth = c(1, 3, 5),
  n.minobsinnode = c(2, 5, 8),
  bag.fraction = c(.5, .75, 1),
  optimal_trees = 0,
  min_RMSE = 0
)

# grid search
for(i in 1:nrow(hyper_grid)) {

  # reproducibility
  set.seed(123)

  # training model
  gbm.tune <- gbm(
    formula = totalBMD ~ .,
    distribution = "gaussian",
    data = treeDB[train,],
    n.trees = 100,
    interaction.depth = hyper_grid$interaction.depth[i],
    shrinkage = hyper_grid$shrinkage[i],
    n.minobsinnode = hyper_grid$n.minobsinnode[i],
    bag.fraction = hyper_grid$bag.fraction[i],
    train.fraction = .75
  )
```

```
  # adding min training error and trees to grid
  hyper_grid$optimal_trees[i] <- which.min(gbm.tune$valid.error)
  hyper_grid$min_RMSE[i] <- sqrt(min(gbm.tune$valid.error))
}

#checking best fits
hyper_grid %>%
  dplyr::arrange(min_RMSE) %>%
  head(10)

#fine-tuning hyper-parameter based on previous results
hyper_grid_fine <- expand.grid(
  shrinkage = c(.1, .2, .3),
  interaction.depth = c(3,5,7),
  n.minobsinnode = c(2,3,4,5),
  bag.fraction = c(.5,.625,.75),
  optimal_trees = 0,               # a place to dump results
  min_RMSE = 0                     # a place to dump results
)

# new grid search
for(i in 1:nrow(hyper_grid_fine)) {

  # reproducibility
  set.seed(123)

  # training model
  gbm.tune <- gbm(
    formula = totalBMD ~ .,
    distribution = "gaussian",
    data = treeDB[train,],
    n.trees = 100,
    interaction.depth = hyper_grid_fine$interaction.depth[i],
    shrinkage = hyper_grid_fine$shrinkage[i],
    n.minobsinnode = hyper_grid_fine$n.minobsinnode[i],
    bag.fraction = hyper_grid_fine$bag.fraction[i],
    train.fraction = 0.75
  )

  # adding min training error and trees to grid
  hyper_grid_fine$optimal_trees[i] <- which.min(gbm.tune$valid.error)
  hyper_grid_fine$min_RMSE[i] <- sqrt(min(gbm.tune$valid.error))
}

#checking new best fits
hyper_grid_fine %>%
  dplyr::arrange(min_RMSE) %>%
  head(10)

#building single model with the identified parameters
set.seed(123)
boost.fit<-gbm(totalBMD~.,
```

```
            distribution="gaussian",
            data=treeDB[train,],
            n.trees=50,
            interaction.depth = 3,
            n.minobsinnode = 3,
            bag.fraction=0.75,
            shrinkage=0.3,
            cv.folds=5)

#checking model results
boost.fit

#plotting model performance
png(filename = "cvError.png",width=1600,height=1600,units="px",res=300)
gbm.perf(boost.fit, method = "cv")
dev.off()

#plotting relative importance of variables
png(filename = "relativeImportance.png",width=1600,height=1600,units="px",res=300)
par(mar = c(5, 8, 1, 1))
vip::vip(boost.fit,n.trees=50,num_features=9)+theme_classic()+ylab("Relative influence (%)")
dev.off()

#predicting risk in test data to include them in the database
pred<-predict(boost.fit,n.trees=50,newdata=treeDB[-train,])

#loading database with the predicted and actual BMD values and its correspondent Z-score
load("predMat.RData")

#calculating root mean squared error
sqrt(mean((predMat$realBMD-pred)^2))

#showing the accuracy of the prediction
table(real=!predMat$realAdjZ>-1,pred=!predMat$predAdjZ>-1)

#building single tree for practical application
indTreeDB<-renacimiento[,c(38,2:9,30:37)]
indTreeDB<-indTreeDB[random_index,]
indTree<-rpart(risk~height+weight+finaPoints+handgrip+swimYears+time30m+time50m+BMI+age,
            data=indTreeDB[train,],method="class",minsplit=20,
            parms=list(loss=matrix(c(0,1,1,0),byrow=TRUE,nrow=2)))

#plotting contents of the individual tree
png(filename = "individualTree.png",width=1600,height=1600,units="px",res=300)
rpart.plot(indTree,box.palette=gray(seq(.6, 1, length.out=9)),type=5,extra=0)
dev.off()

#calculating the prediction on the remaining 30% of the sampling
indPred<-predict(indTree,newdata=indTreeDB[-train,])[,2]>1/2

#showing the accuracy of the prediction
table(real=!predMat$realAdjZ>-1,pred=indPred)
```
