# Supplementary material for: Design of a Computer Model for the Identification of Adolescent Swimmers at Risk of Low BMD
Source: Int J Environ Res Public Health. 2023 Feb 16;20(4):3454. doi: 10.3390/ijerph20043454 (PMC9964481; doi:10.3390/ijerph20043454)
Supplement: Supplementary file 1 [file ijerph-20-03454-s001.zip › Supplementary Table S1.pdf]

Supplementary Table S1. Summary of the variables included in the regression and decision trees.

Variable type	Variable names
Predicted outcome:	Subtotal (whole body less head) bone mineral density (BMD)
Physical fitness:	**Speed**: Time to complete a 30-m sprint run<br>**Handgrip strength**: Sum of best attempts with both arms<br>Lower-limb strength: Distance in a standing long jump<br>Endurance: VO_{2max} estimated from 20-m shuttle-run test
Swimming training and performance:	Weekly training hours, **years of swimming history**, **time in 50-m swim**, **FINA points**
Potential confounders:	**Age**, sex, Tanner stage, **height**, **weight**, **BMI**, calcium intake, participation in other sports

Abbreviations: BMD: bone mineral density; BMI: body mass index; VO_{2max}: maximal oxygen uptake; FINA: International Swimming Federation.

The variables highlighted in bold were statistically significant within the ensemble model and were therefore used to construct the individual decision tree.
